# Two Paediatric Patients with Encephalopathy and Concurrent COVID-19 Infection: Two Sides of the Same Coin?

**DOI:** 10.1155/2021/6658000

**Published:** 2021-03-24

**Authors:** Katerina Vraka, Dipak Ram, Siobhan West, Wei Yen Evelyn Chia, Praveen Kurup, Gayathri Subramanian, Hui Jeen Tan

**Affiliations:** ^1^Department of Paediatric Neurology, Rotal Manchester Children's Hospital, Manchester M13 9WL, UK; ^2^Department of Paediatric Intensive Care Unit, Rotal Manchester Children's Hospital, Manchester M13 9WL, UK

## Abstract

There is increasing evidence that SARS-CoV-2 has neurotropic potential. We report on two paediatric patients who presented with encephalopathy during COVID-19 illness. Both patients had ADEM-like changes in their neuroimaging, negative SARS-CoV-2 RNA PCR in CSF, and paucity of PIMS-TS laboratory findings. However, the first patient was positive for serum MOG antibodies with normal CSF analysis, and the second had negative MOG antibodies but showed significant CSF lymphocytic pleocytosis. We concluded that the first case was a typical case of demyelination, which could have been triggered by different cofactors. In the second case, however, we postulated that the encephalopathic process was triggered by SARS-CoV-2, as no other cause was identified. With these two contrasting cases, we provide evidence that SARS-CoV-2-associated encephalitis can show ADEM-like changes, which can present during the postinfectious phase of COVID-19 illness. As ADEM is a relatively common type of postinfectious encephalitis in children, the distinguishing line between the two conditions of encephalitis and ADEM can be relatively fine. The development of more reliable diagnostic tools (e.g., anti-SARS-CoV-2 antibodies in CSF) might play an assisting role in the differentiation of these encephalopathic processes.

## 1. Introduction

Although the primary target of SARS-CoV-2 is the respiratory system, neurological manifestations have been reported in affected patients [1]. The first case of SARS-CoV-2 meningoencephalitis was reported in March 2020 with a positive specific SARS-CoV-2 RNA in the cerebrospinal fluid (CSF). Since then, there have been multiple reports of SARS-CoV-2-associated encephalitis, with only few showing viral detection in the CSF. Here, we report two contrasting cases of neuroinflammatory involvement of SARS-COV-2 infection in children with absence of any respiratory symptoms.

## 2. Case 1

**Figure 1:**
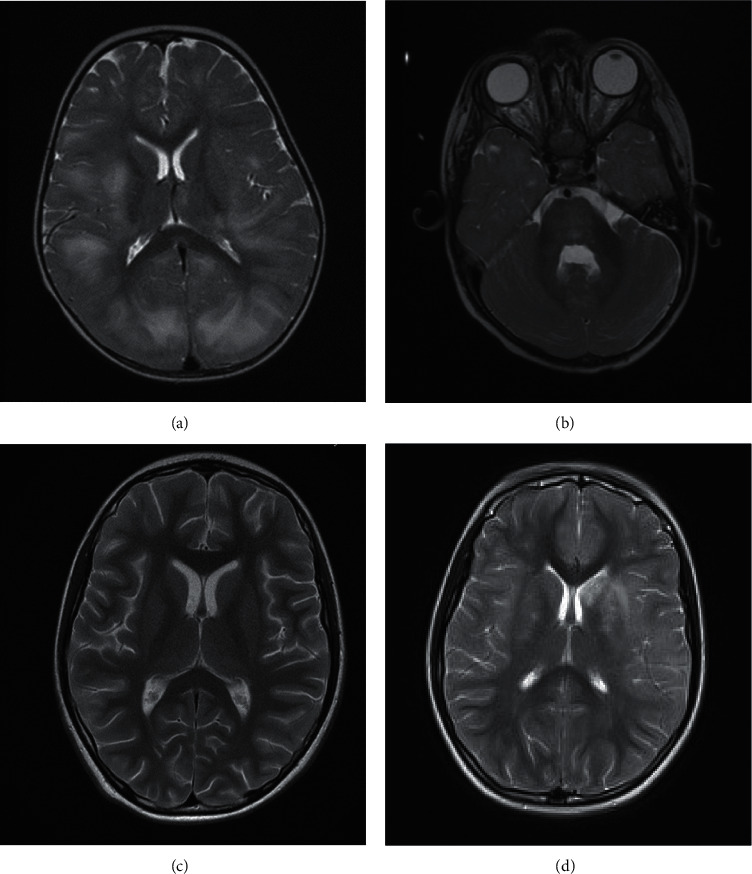
(a, b) MRI brain of Case 1 showing bilateral T2 hyperintensities of the subcortical white matter of all the brain and splenium of the corpus callosum with associated diffusion restriction and signal change in thalami and pons. (c) Case 2: normal MRI brain on acute presentation. (d) Repeat MRI brain 50 days later showing bilateral T2 hyperintensity of the basal ganglia and parasagittal frontal lobes, anterior limb of the left internal capsule, insula, and subcortical white matter regions.

A previously healthy 13-month-old girl presented at a local hospital with altered consciousness, seizures, and a 3-day history of fever. She had received her MMR vaccination a month prior and had a febrile illness ten days before presentation. On admission, her pupils were equal and bilaterally reactive to light; she had no cranial nerve palsies, but she had decorticate posturing and a Glasgow Coma Scale of 5. She required intubation, intravenous antibiotics, aciclovir, fluid boluses, and levetiracetam and was transferred to our Critical Care Unit. She remained ventilated for 4 days and developed hypertension, needing treatment. Due to episodes of episodic right-hand twitching, she had an EEG, which showed diffuse slow-wave background activity, in keeping with encephalopathy, but no epileptiform discharges. Nasopharyngeal (NPA) PCR was initially positive for SARS-CoV-2 and adenovirus, but only SARS-CoV-2 remained positive for the following 2 weeks. CSF analysis showed 10/mm^3^ white cells, being negative for SARS-CoV-2 RNA (Table 1). CT head showed bihemispheric white matter hypodensities, posteriorly more than anteriorly, and an initial suspicion of posterior reversible encephalopathy syndrome was raised. The subsequent MRI brain, on day 4 of admission, showed bilateral widespread white matter high-signal abnormalities, including the splenium of the corpus callosum with associated diffusion restriction and high signal in the thalami and pons (Figures 1(a) and 1(b)). This was initially felt to be consistent with COVID-19 encephalopathy, especially given the presence of the splenial lesion [2, 3]. Spinal MRI was normal. However, she was treated clinically with steroids for acute disseminated encephalomyelitis (ADEM). Her serum myelin oligodendrocyte glycoprotein (MOG) antibody result was subsequently detected (Table 1). She continued to respond well to the steroid therapy. She initially presented drowsy, with poor central tone and unsafe swallow. Progressively, she became increasingly alert and her tone improved. On discharge, she was able to sit, took a few steps, clapped with songs, recognised voices, and was able to eat and drink normally. However, she was not fixing and following consistently, and after a normal ophthalmic examination, it was presumed that she had cortical visual impairment. Four months following her presentation, her vision had improved, she was walking, and she showed good developmental progress, with no further seizures on levetiracetam.

## 3. Case 2

A previously healthy 10-year-old girl presented at a district hospital with vomiting, lethargy, and a 2-day history of pyrexia. Six days before, she had developed ageusia, headache, and malaise, and on day 3, she had a positive NPA PCR for SARS-CoV-2. On day 9, she developed fluctuating sensorium and urinary incontinence. On day 10, she stopped speaking, mobilising, and using her right arm, showing hypertonia, brisk reflexes, right-sided Babinski, and sluggish pupils. She received intravenous aciclovir and antibiotics, had a normal CT head, and was transferred to our Critical Care Unit. On day 11, she was noted to have autonomic disturbance with hypertension and was intubated for neuroprotection for suspected raised intracranial pressure. MRI brain and spine, on day 12, were unremarkable (Figure 1(c)), but CSF analysis showed a markedly raised white cell count (WCC) of 6075/mm^3^ with 93% lymphocytes and CSF protein of 0.58 g/L. CSF SARS-CoV-2 RNA test was negative. There were no clinical and laboratory findings in keeping with a diagnosis of paediatric inflammatory multisystem syndrome temporally associated with COVID-19 (PIMS-TS), i.e., absence of multisystem involvement clinically and no signs of inflammation on laboratory or radiological tests [4]. MOG, NMDA receptor, and VGKC antibodies were negative (Table 1). She was extubated on day 13 and, a week later, improved to her normal abilities apart from fatiguability and neglect of her right arm. SARS-CoV-2 NPA PCR remained positive for 30 days. She was discharged from the hospital, but due to her persistent right-arm neglect, MRI neuroaxis, lumbar puncture, EEG, and MOG antibody serology were repeated 50 days after her illness onset. Repeat MRI brain (Figure 1(d)) showed asymmetric bilateral high-signal lesions in the basal ganglia and the subcortical white matter in the frontal and temporal lobes, with involvement of the left internal capsule and left hippocampus. MRI orbits and spine, repeated CSF analysis, MOG antibodies (Table 1), and EEG were unremarkable. Two months later, she was well, with some neglect of her right upper limb, and concerned around her verbal memory, awaiting formal neurocognitive assessments.

## 4. Discussion

SARS-CoV-2 uses the angiotensin-converting enzyme 2 (ACE2) receptor for entry and the serine protease TMPRSS2 for S protein priming [5]. Recent studies demonstrate that SARS-CoV-2 exhibits neurotropic properties [6, 7]. A direct neuroinvasive effect of the virus could be explained by its retrograde movement along the olfactory or the peripheral lung nerves to the central nervous system (CNS) or via haematogenic migration through the CNS endothelia that express ACE2 receptors. An indirect effect can result from the leakage of inflammatory mediators through a permeable blood-brain barrier [8].

Neither of our patients, interestingly, had respiratory symptoms or signs of PIMS-TS. Case 1 had a splenial lesion, which appears to be a consistent finding in children with PIMS-TS [2]. ADEM is a rare immune-mediated demyelinating disease that can be triggered by viruses or vaccines and dominated by an encephalopathic picture. It is important to consider COVID-19 infection in children with ADEM-like illnesses, even in the absence of respiratory symptoms or signs of PIMS-TS.

The coexistence of encephalopathy, seizures, MOG antibodies, and the typical MRI findings in Case 1 supports the diagnosis of a demyelination process related to MOG antibodies. As this child had a concurrent adenovirus infection on presentation, with a recent history of MMR vaccination, it is difficult to establish whether the trigger that initiated the neuroinflammatory process was COVID-19 infection alone or a combination of these factors. The consequence of having MOG-positive antibodies is a risk of relapsing demyelination and may have different implications for long-term management.

The temporal sequence of signs of encephalopathy and neurological symptoms, in a known COVID-19 infection, along with the later MRI brain changes in Case 2, is in keeping with a neuroinflammatory process triggered by SARS-CoV-2. Lymphocytic pleocytosis (with a WCC of fewer than 100 cells/mm^3^) is common in ADEM [9], but our case noted unusually high numbers. Higher cell counts have been reported in patients with PIMS-TS and encephalopathy [2]. For this reason, it was important to perform a full screen to exclude other causes.

Reported cases of COVID-19 acute encephalitis did not show such a marked pleocytosis in the CSF, and it was common to see MRI changes during the acute phase of the encephalopathic illness rather than during the recovery phase [1]. The absence of MOG antibodies, the lymphocytic pleocytosis, and the ADEM-like changes could suggest that SARS-CoV-2 may have triggered a neuroinflammatory process in this child.

The SARS-CoV-2 CSF RNA test was negative in both cases, but we acknowledge that this is not a validated test. The detection of anti-SARS-CoV-2 antibodies in CSF could support the diagnosis of patients with COVID-19 encephalitis, but this is currently unavailable in our centre [10].

In conclusion, although SARS-CoV-2 can trigger an inflammatory process mimicking ADEM, it is important to exhaust all means of investigations before labelling an encephalopathy as acute COVID-19 encephalitis.

## Figures and Tables

**Table 1:** Salient laboratory findings of presented cases.

	Case 1	Case 2	—
	Acute presentation	Acute presentation	50 days later
CRP (max)	13	8	—
ESR (max)	—	37	—
Coagulation	Normal	Normal	—
Fibrinogen	4	3.5	—
d-Dimer	354	859	—
Troponin	—	Normal	—
Ferritin	172	209	—
Pro-BNP (max)	83	591	—
Vitamin D	155	31	—
Serum lactate (mmol/L)	1.5	1.2	1.7
Blood cultures	Negative	Negative	—
Viral serology (IgG/IgM)	—	EBV IgG positive/IgM negative	—
		CMV IgG positive/IgM negative	
	CMV PCR negative	—	—
SARS-CoV-2 NPA PCR	Positive for 3 weeks	Positive for 4 weeks	Negative
Other NPA PCR virology	Adenovirus positive	No	—
Mycoplasma PCR	Negative	Negative	—
MOG IgG antibodies	Positive	Negative	Negative
NMDA rec. antibodies	—	Negative	Negative
VGKC antibodies	—	—	Negative
Vasculitis screen (including C3, C4, ANA, rheumatoid factor, IgG dsDNA, MPO and PR3, and cardiolipin profile)	—	Negative	—
Immunoglobulin profile	Normal	Normal	—
CSF WCC (cells/mm^3^)	10	6075	—
93% lymphocytes	5	—	—
CSF RCC (cells/mm^3^)	255	25	10
CSF protein (g/dL)	0.31	0.58	0.48
CSF glucose (mmol/L)	4.7	4.8	3
CSF lactate (mmol/L)	1.4	1.9	1.7
CSF organisms	No	No	No
CSF bacterial culture	Negative	Negative	Negative
CSF virology including SARS-CoV-2 RNA	Negative	Negative	Negative
CSF oligoclonal bands (paired with serum)	Normal	—	Normal

NPA: nasopharyngeal aspirate; MOG: myelin oligodendrocyte glycoprotein; WCC: white cell count; RCC: red cell count; CSF: cerebrospinal fluid.

## Data Availability

The data used to support the findings of these case reports are included within the table and figures of this article.
